# Sociodemographic Factors Associated with Request for Labor Epidural Analgesia in a Tertiary Obstetric Hospital in Vietnam

**DOI:** 10.1155/2021/8843390

**Published:** 2021-01-30

**Authors:** Lam D. Nguyen, Anh D. Nguyen, Michaela K. Farber, Chi T. Phan, Luong T. Khuat, Ha T. Nguyen, Tuan M. Dang, Ha T. Ngoc Doan

**Affiliations:** ^1^Hanoi Medical University, Hanoi 100000, Vietnam; ^2^Hanoi Obstetrics and Gynecology Hospital, Hanoi 100000, Vietnam; ^3^Brigham and Women's Hospital, Boston, MA 02115, USA; ^4^Vietnam National University, Hanoi 100000, Vietnam; ^5^Institute for Preventive Medicine and Public Health, Hanoi Medical University, Hanoi 100000, Vietnam

## Abstract

This study is aimed at examining the sociodemographic factors associated with the utilization of labor epidural analgesia at a large obstetric and gynecology hospital in Vietnam. This was a cross-sectional study of women who underwent vaginal delivery in September 2018 at the Hanoi Obstetrics and Gynecology Hospital. The utilization of epidural analgesia during labor was determined. Univariate and multivariate regression models were applied to evaluate the association between patient demographic and socioeconomic factors and request for labor epidural analgesia. A total of 417 women had vaginal deliveries during the study period. 207 women utilized epidural analgesia for pain relief during labor, and 210 did not. Parturients older than 35 years of age (OR 2.84, 95% CI 1.11-8.17), multiparous women (OR 2.8 95% CI 1.85-4.25), women living from an urban area, women with higher income (OR 6.47, 95% CI 2.59-19.23), and women with higher level of education were more likely to utilize labor epidurals. Factors related to a parturient request for epidural analgesia during labor at our tertiary obstetric hospital included age greater than 35 years, multiparity, and high income and education levels. Educational outreach to women about the benefits of epidural analgesia can target women who do not share these demographic characteristics.

## 1. Introduction

Epidural analgesia is an effective and increasingly common method for pain relief during labor [1, 2]. This technique has been widely used in developed countries, whereas its use in developing countries such as Vietnam is less common. The use of epidural analgesia in the US tripled between 1981 and 2001, and up to 86% of women utilized this technique in university hospitals [3–6]. In the past few years, this method has been widely deployed in Vietnam. However, the epidural technique has not been uniformly covered by Vietnamese health insurance, and many women remain unaware of this option. Determinants of current requests and utilization of epidural analgesia among Vietnamese women during labor are unknown. Therefore, this study is aimed at identifying patient factors associated with request and utilization of labor epidural analgesia at an obstetric hospital in Hanoi, Vietnam.

## 2. Materials and Methods

The study protocol was approved by the Scientific and Ethical Committee of Hanoi Obstetrics and Gynecology Hospital. Written informed consent was obtained from all participants prior to the interview and data collection.

A retrospective cross-sectional study was conducted to evaluate women who underwent vaginal delivery during the month of September 2018 at the Obstetrics and Gynecology Hospital in Hanoi, Vietnam. Patients were grouped based on using epidural analgesia or not during admission for labor and delivery. Demographic variables collected included maternal age (classified by age less than 18 years, 18-34 years, 35-45 years, and >45 years), residential area (urban or rural), ethnicity (Kinh, the largest ethnic group in Vietnam, or alternative ethnic minority), education levels (primary, secondary, high school, and graduate school), occupation, health insurance, level of health knowledge, and income. Data were collected by in-person interviews 24 hours after delivery and in a standardized way by coinvestigators of this study.

Descriptive and analytical statistics were performed using SPSS 20.0 software. Proportions of variables and corresponding 95% confidence interval (CI) were calculated. Multivariate logistic regression was performed to examine the probability of epidural analgesia use in relation to sociodemographic data obtained. A significance level of *p* < 0.05 was applied.

## 3. Results and Discussion

A total of 417 women who underwent vaginal delivery in September 2018 were identified. 207 patients utilized epidural analgesia during labor, and 210 did not. Sociodemographic and obstetric characteristics of the participants are shown in Tables 1 and 2. All sociodemographic, other than ethnicity, employment, and health insurance status, and obstetric variables were different between those that used epidural analgesia and those that did not.

### 3.1. Sociodemographic and Obstetric Characteristics of Parturients

Our study demonstrates that women with health insurance and higher education levels are more likely to request epidural analgesia during labor. These findings were consistent with a previous study in the United States [7]. A research conducted by Koteles et al., which evaluated the sociodemographic and obstetric factors associated with epidural analgesia during labor on 5350 Canadian women, revealed that all sociodemographic and obstetric variables were statistically significantly different between two groups [6].

### 3.2. The Main Concerns about Epidural Analgesia during Labor

The findings of the current research suggested that the parturients in the epidural analgesia group were more worried about pain in labor than the ones in the no-epidural analgesia group (*p* < 0.05). These are similar to the study of Koteles et al. in which the majority of women who used epidural analgesia (62–64%) had at least one stressful period prior to the birth of their child, suggesting that pregnancy could be stressful in many women's life [6].

In our study, there were many parturients in both the epidural analgesia group and nonepidural analgesia group worried about the side effects of epidural analgesia during the labor (64.7% vs. 65.7%, respectively). In Vietnam, there have been still many misconceptions and fears of epidural analgesia use. These misconceptions were also recorded in the study of Koteles et al. (15.9% women believed that the epidural analgesia causes paraplegia) [6]. In other studies, most of women in Pakistan (Karachi) and Hong Kong believed that epidural analgesia could result in permanent backache which could cause muscle weakness in the mothers' lower limbs during labor [8–10].

Many studies showed that a lack of knowledge about epidural analgesia and its side effect discouraged many women from using it. It was recommended that thorough information about epidural analgesia should be provided for women in prenatal courses. Otherwise, due to the remaining concerns of side effects and unavailability of epidural services, several alternative nonpharmacological methods of pain relief could also be considered, including labor support by family members, hypnobirthing (the practice of self-hypnosis and breathing at birth), acupuncture and acupressure, or water birth.

Our institute has more experience with labor supporters and hypnobirthing than the remaining methods. Labor supported by family members, usually a husband, is regularly offered in our institute for all parturients requiring delivery rooms with higher quality (accounted for approximately 40% of all deliveries in our hospital). The husband should stand next to his wife and encourages her while she is laboring and delivering. Its sole effectiveness of pain relief is insignificantly observed.

Hypnobirthing is occasionally used in our hospital, but at a very low rate. This service is provided by a trained midwife. All pregnant women attending available prenatal courses in our hospital will be consulted about this method. Participants who want to use this method at birth can attend the related prenatal classes to learn how to breathe and undergo self-hypnosis at least 1-2 months before their estimated time of delivery. While delivering, the parturient should practice it following the instructions of a trained midwife. However, a few parturients choose to use this technique solely and their pain is slightly relieved as in our observation.

### 3.3. Factors Associated with the Parturient's Choice of Epidural Analgesia during Labor

Data from Table 3 using multiple logistic regression analysis of factors associated with a parturient receiving epidural analgesia during labor revealed that several socioeconomic factors helping to predict the patient epidural choice were women over 35 years, multiparity, higher income, getting a high level of education, living in urban areas, housewives, or office workers. Other studies confirmed our findings that socioeconomic factors were associated with epidural use. One study by Hueston et al. on 8229 deliveries at five hospitals in the United States showed that epidural analgesia during labor was associated with the increasing maternal age, Caucasian ethnicity, and private insurance coverage [11].

In our study, the parturients over 35 years old and multiparous women were likely to use epidural analgesia. This could be explained by the fact that almost these parturients previously had an epidural experience, so it was easier for them to decide to choose this method for their next delivery.

Another factor associated with the proportion of epidural analgesia use in our study was the education level of the women. The women with higher education levels were likely to use epidural analgesia (for those who graduated from college or university, the rate of choice for epidural anesthesia was 4.02 times higher than that of women who completed primary school only, 95% CI: 2.62-6.18). There were similarities between the findings from our study and other international research evidence. The study of Glance et al. in the US reported that the labor epidural analgesia rate was 22.6% in women with <8^th^ grade education versus 48.1% in those who finished high school [12]. A study of Sheiner et al. from Israel showed that the rate of requesting an epidural was only 4.9% in women with <5 years of schooling, compared with 84% of those with academic school education [13]. Some previous studies reported similar findings [4, 14, 15]. This may explain that the highly educated pregnant women would better understand the physiological mechanism of labor and have more information about the methods of pain relief during labor, so it was easy for them to choose this method. Another probable explanation was that Vietnamese women and Asian women, in general, considered labor pain natural and would like to tolerate it. Therefore, visiting health providers for prenatal health courses and attendance to prenatal health courses are very important to improve their knowledge and to change their attitudes and decisions. Similar findings were reported by Alakeely et al., in which education on epidural analgesia during antenatal care is needed for better decision making regarding the use of epidural analgesia during labor [16].

Pregnant women are sometimes also likely to claim on health insurance of consulted pain relief methods. We always inform the prenatal women that while popular methods of pharmacological pain relief such as morphine are covered by the health insurance, epidural analgesia is not yet covered, since it has only been practiced in major hospitals in Vietnam for recent years. This partly results in socioeconomic inequalities in health care, which are already a problem in Vietnam [17]. Low-income and rural parturients in our study demanded epidural analgesia less than high-income and urban women since they are less likely to pay for this service. Fewer parturients in the countryside accept epidural anesthesia due to medical costs; rural women often endure pain better, and in some places with misconception that pain at giving of birth is normal, they try to tolerate and suffer from this pain without looking for pain relief.

According to Harkins et al., the opinion of the husband was a key factor affecting the request of epidural use. Therefore, prenatal courses for all pregnant women are needed to help both spouses making their decisions [7].

Despite its novelty and scientific significance, our research had certain limitations. Firstly, due to the 24-hour postpartum interview, some parturients did not remember the details of the birth; secondly, because the research was conducted only at the Hanoi Obstetrics and Gynecology Hospital, which is the leading obstetric hospital in Hanoi and teaching hospital, the study subjects were mainly pregnant women living in Hanoi areas, so they did not represent the pregnant women in Vietnam. The Vietnamese women who gave birth in nonuniversity public hospitals or in small maternity units sometimes cannot achieve epidural analgesia because of a lack of available anesthesiologists on the spot. Similar findings were reported by Liu et al., Ogboli-Nwasor et al., and Le Ray et al. [14, 18, 19].

## 4. Conclusions

In summary, the probable factors related to the choice of epidural anesthesia during labor among vaginal delivery women at the Hanoi Obstetrics and Gynecology Hospital, Vietnam, were multiparous women, women over 35 years old, living in the urban area, and having high income and high education.

## Figures and Tables

**Table 1:** Univariate analysis of sociodemographic and obstetric characteristics of parturients.

Characteristics		Epidural (*N* = 207)		No epidural (*N* = 210)		*p* value
		*n*	**%**	*n*	**%**	
Age (years)	<18	1	0.5	4	1.9	0.02
	18-35	199	96.1	187	89.0	
	36-45	7	3.4	19	9.0	

Parity	Nulliparous	129	62.3	78	37.1	<0.01
	Multiparous	78	37.7	132	62.9	

Education	Primary	11	5.3	37	17.6	<0.01
	Secondary	18	8.7	54	25.7	
	High school	35	16.9	44	21.0	
	Graduate	143	69.1	75	35.7	

Ethnicity	Minorities	11	5.3	20	9.5	0.101
	Kinh	196	96.7	190	90.5	

Residence	Urban	178	86.0	130	61.9	<0.01
	Rural	29	14.0	80	38.1	

Income	Low	6	2.9	34	16.2	<0.01
	High	201	97.1	176	83.8	

Occupation	Homemaker	43	20.8	13	6.2	<0.01
	Farmer	3	1.4	47	22.4	
	Worker	17	8.2	82	39.0	
	White-collar workers	144	69.6	68	32.4	

**Table 2:** Patient concerns about epidural analgesia during labor.

Characteristics		Epidural (*N* = 207)		No epidural (*N* = 210)		*p* value
		*n*	**%**	*n*	**%**	
Health insurance status	No health insurance coverage	50	24.2	66	31.4	0.10
	Health insurance coverage	157	75.8	144	68.6	

Epidural-related concerns	Pain in labor	185	89.4	111	52.9	<0.01
	Side effects	134	64.7	138	65.7	0.83
	Expense	18	8.7	101	48.1	<0.01

**Table 3:** Multiple logistic regression analysis of factors associated with parturient choice of epidural analgesia during labor.

Characteristics		OR	95% CI	*p* value
Age group (year)	≤35	Ref		
	>35	2.84	1.11–8.17	0.02

Parity	Nulliparous	Ref		
	Multiparous	2.80	1.85–4.25	<0.01

Education	Primary	Ref		
	Secondary	0.28	0.15–0.50	<0.01
	High school	0.77	0.45–1.29	0.29
	Graduate	4.02	2.62–6.18	<0.01

Ethnicity	Minorities	Ref		
	Kinh	1.88	0.83–4.45	0.10

Residence	Urban	Ref		
	Rural	0.26	0.16–0.44	<0.01

Income	Low	Ref		
	High	6.47	2.59–19.23	<0.01

Employment status	Unemployed	Ref		
	Employed	0.78	0.51–1.21	0.25

Occupation	Farmer	Ref		
	Homemaker	3.97	2.01–8.31	<0.01
	Worker	0.14	0.07–0.25	<0.01
	Officer	4.77	3.09–7.38	<0.01

Health insurance status	No health insurance	Ref		
	Health insurance	1.44	0.91–2.27	0.10

## Data Availability

The raw data and syntax used to support the findings of this study are available from the corresponding author upon request.
